# Cardiovascular Disease Mortality in Mississippi, 2000–2018

**DOI:** 10.5888/pcd19.210385

**Published:** 2022-02-24

**Authors:** Vincent L. Mendy, Tawandra Rowell-Cunsolo, Meghan Bellerose, Rodolfo Vargas, Byambaa Enkhmaa, Lei Zhang

**Affiliations:** 1 Department of Epidemiology and Biostatistics, School of Public Health, College of Health Sciences, Jackson State University, Jackson, Mississippi; 2 Sandra Rosenbaum School of Social Work, University of Wisconsin-Madison, Madison, Wisconsin; 3 Columbia University Mailman School of Public Health, New York, New York; 4 Office of Health Data and Research, Mississippi State Department of Health, Jackson, Mississippi; 5 Department of Internal Medicine, School of Medicine, University of California, Davis, California; 6 School of Nursing, University of Mississippi Medical Center, Jackson, Mississippi

## Abstract

**Introduction:**

Cardiovascular disease (CVD) is the leading of cause of death in Mississippi. We explored trends in CVD death rates among adults in Mississippi aged 35 years or older to assess changes from 2000 through 2018.

**Methods:**

We extracted data from Mississippi Vital Statistics from 2000 through 2018. We used underlying cause-of-death codes from the *International Classification of Diseases, Tenth Revision* (ICD-10) to identify CVD deaths; we included all cases with codes I00–I09, I11, I13, I20–I51, I60–I69, and I70. We calculated age-adjusted CVD death rates for the overall population by age, race, sex, and race-by-sex groups.

**Results:**

Overall, the age-adjusted CVD death rate declined from 832.3 deaths per 100,000 population in 2000 to 550.5 deaths per 100,000 in 2018, a relative decline of 33.9% and an average annual decline of −2.3% (95% CI, −2.7% to −1.8%). Age-adjusted CVD death rates declined from 2000 through 2018 for all groups, but the magnitude of decline varied by subgroup (men, −2.0%; women, −2.6%; non-Hispanic Black, −2.4%; non-Hispanic White, −2.2%; non-Hispanic Black women, −3.0%; non-Hispanic White women, −2.5%; non-Hispanic Black men −2.1%; non-Hispanic White men −2.0%). Age-specific analysis indicated a significant average annual increase of 1.7% (95% CI, 0.6%–2.9%) from 2011 through 2018 for the group aged 55 to 64 years.

**Conclusion:**

From 2000 through 2018, age-adjusted CVD death rates in Mississippi declined for all age/race/sex groups. However, the magnitude of decline varied by subgroup. Targeted interventions for CVD risk reduction are needed for adults aged 55 to 64 years in Mississippi, the only age group in which we observed a significant annual increase in CVD death rates.

**Summary**

**What is already known on this topic?**

Cardiovascular disease (CVD) is the leading of cause of death in Mississippi.

**What is added by this report?**

Age-adjusted CVD death rates declined from 2000 through 2018 for all groups studied (men, women, Black, White, Black women, White women, Black men, White men), but the magnitude of decline varied among subgroups. Age-specific analysis indicated a significant annual increase of 1.7% during 2011–2018 for the group aged 55 to 64 years.

**What are the implications for public health practice?**

Targeted interventions for CVD risk reduction are needed for adults aged 55 to 64 years in Mississippi.

## Introduction

Cardiovascular disease (CVD) is the leading of cause of death in Mississippi (1), accounting for 29.7% of all deaths in 2018 (1). In 2017, Mississippi had the highest mortality rates of heart disease and the second-highest mortality rates of stroke in the US (2). A previous study indicated increases in CVD mortality and marked CVD disparities during 1979–1995 in Mississippi (3).

Beginning in 2008, to better monitor CVD risk factors at a regional level in areas with a high burden of CVD, the Mississippi State Department of Health, in collaboration with the Centers for Disease Control and Prevention, developed and initiated a Cardiovascular Health Examination Survey in the Mississippi Delta region (4). The Mississippi State Department of Health also implemented initiatives, including the Mississippi Mayors’ Health Councils program, the Delta Alliance for Congregational Health, and the Barbers Reaching Out To Help Educate on Routine Screening (BROTHERS) program (5), that aimed at promoting heart-healthy lifestyle choices and reducing CVD risk factors in the 18-county Mississippi Delta region (6).

We recently reported that the age-adjusted heart disease (7) and stroke (8) mortality rates among Mississippi adults declined significantly during 1980–2013 and 2000–2016, respectively. Although our research identified trends in heart disease and stroke mortality rates, other research suggests that additional cardiovascular conditions substantially contribute to CVD-related mortality (9). More research is needed to better understand the extent to which Mississippi has experienced reductions in all CVD-related mortality. Understanding trends in CVD mortality as well as the racial and race–sex-specific dipartites in CVD mortality is important to assessing the impact of previous and ongoing CVD interventions, which could affect future public health policies in Mississippi. To assess changes in CVD mortality in Mississippi in recent years, we evaluated trends and disparities in CVD mortality among Mississippi adults aged 35 years or older from 2000 through 2018. We calculated the annual percentage change (APC, trend segment) and the average annual percentage change (AAPC) in age-adjusted death rates from 2000 through 2018 among Mississippi adults aged 35 years or older and examined the differences in the AAPC by sex, age, and race groups.

## Methods

We extracted data on the number of deaths due to CVD among adults aged 35 years or older for each year from 2000 through 2018 from Mississippi Vital Statistics (1). In 2018, most (98.9%) deaths due to CVD in Mississippi occurred among people aged 35 years or older (1); therefore, we included only decedents aged 35 years or older in our analysis. We used underlying cause-of-death codes from the *International Classification of Diseases, Tenth Revision* (ICD-10) to identify CVD deaths; we included all cases with codes I00–I09, I11, I13, I20–I51, I60–I69, and I70 (10). We then used the related US census estimates for the Mississippi population to calculate the crude and age-specific CVD death rates and standard errors for the overall population, by age group (35–54, 55–64, 65–74, 75–84, and ≥85 y), race (non-Hispanic Black or non-Hispanic White [hereinafter, Black or White]), sex (male or female), and race and sex (Black male, Black female, White male, or White female) for each year by using SAS version 9.4 (SAS Institute Inc).

We adjusted death rates by using the 2000 US standard population (11). We then exported age-adjusted CVD death rates and standard errors to the US Surveillance, Epidemiology, and End Results (SEER) joinpoint regression program version 4.7.0.0 (12) to calculate the AAPC in CVD death rates for the overall Mississippi population as well as by race, sex, and a combination of race and sex. We restricted analyses to non-Hispanic Black and non-Hispanic White groups; these racial groups accounted for 96.9% of the state’s population in 2018 (1). Joinpoint regression analysis identifies trend breaks (joinpoints) or points of significant change in a trend. This analysis identifies periods with distinct log-linear trends in CVD death rates (13). Using the Bayesian information criterion to select the most parsimonious model with the best fit, we specified a maximum of 3 joinpoints (12,13). We used the slopes of the models to calculate the APC for each trend segment and the AAPC (the weighted average of the APCs) (12). For each AAPC, we calculated 95% CIs and considered them significantly different from 0 at *P* values <.05. The Jackson State University Institutional Review Board approved the study.

## Results

**Figure 1:**
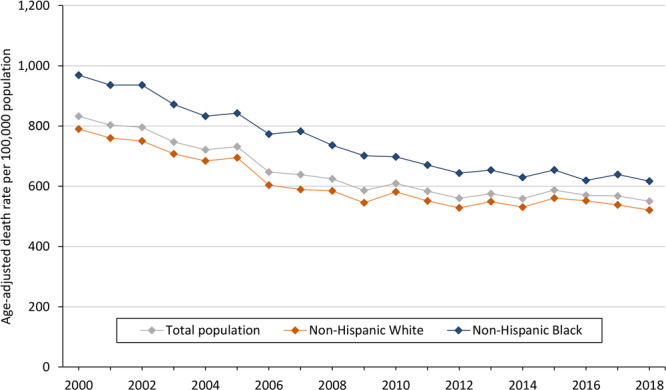
Overall age-adjusted cardiovascular disease death rates per 100,000 population in Mississippi, 2000–2018, by race.

Overall, the age-adjusted CVD death rate declined from 832.3 deaths per 100,000 population in 2000 to 550.5 deaths per 100,000 in 2018, a relative decline of 33.9% and an average annual decline of −2.3% (95% CI, −2.7% to −1.8%) (Figure 1). This period included 2 segments: 2000–2009 and 2009–2018. The overall rate declined significantly by −3.8% (95% CI, −4.5% to −3.2%) per year during 2000–2009, but it did not significantly decline (APC, −0.7; 95% CI, −1.4% to 0%) during 2009–2018 (Table).

**Table:** 

Year	Death rate per 100,000 population		
	Total population	Non-Hispanic White	Non-Hispanic Black
2000	832.3	790.0	969.0
2001	803.5	760.2	936.1
2002	795.9	750.1	936.1
2003	747.1	707.5	871.8
2004	721.2	684.3	832.8
2005	731.5	695.1	842.9
2006	647.1	603.9	773.1
2007	638.7	589.2	782.6
2008	624.6	584.8	736.1
2009	586.1	545.4	701.4
2010	609.7	581.5	697.7
2011	583.6	551.3	670.7
2012	560.4	528.2	644.0
2013	575.4	548.9	653.8
2014	559.0	530.8	629.5
2015	587.6	560.7	654.2
2016	570.1	551.9	619.5
2017	568.0	538.4	639.2
2018	550.5	521.0	617.2

**Table:** Trends in Age-Adjusted Cardiovascular Disease (CVD) Death Rates Among Adults Aged ≥35 Years in Mississippi, 2000–2018

Characteristic	No. of CVD deaths (age-adjusted rate)^a^		AAPC (95% CI)^b^	Trend segment 1		Trend segment 2	
	2000	2018	2000–2018	Years	APC^c^ (95% CI)	Years	APC (95% CI)
**Overall**	11,510 (832.3)	9,789 (550.5)	−2.3^d^ (−2.7 to −1.8)	2000–2009	−3.8^d^ (−4.5 to −3.2)	2009–2018	−0.7 (−1.4 to 0)
**Sex**							
Male	5,253 (986.9)	5,154 (684.8)	−2.0^d^ (−2.5 to −1.5)	2000–2009	−3.5^d^ (−4.3 to −2.8)	2009–2018	−0.4 (−1.2 to 0.4)
Female	6,257 (709.6)	4,635 (443.4)	−2.6^d^ (−3.1 to −2.2)	2000–2009	−4.1^d^ (−4.8 to −3.5)	2009–2018	−1.1^d^ (−1.9 to −0.4)
**Age group, y** e							
35–54	1,039 (132.0)	917 (125.0)	−0.5^d^ (−0.9 to −0.2)	2000–2018	−0.5^d^ (−0.9 to −0.2)	—f	—f
55–64	1,263 (513.8)	1,530 (396.5)	−1.1^d^ (−1.6 to −0.5)	2000–2011	−2.8^d^ (−3.4 to −2.2)	2011–2018	1.7^d^ (0.6 to 2.9)
65–74	2,151 (1,158.3)	2,113 (753.1)	−2.4^d^ (−2.9 to −1.9)	2000–2012	−3.8^d^ (−4.3 to −3.3)	2012–2018	0.5 (−0.9 to 2.0)
75–84	3,363 (2,926.3)	2,409 (1,701.9)	−2.9^d^ (−3.4 to −2.3)	2000–2009	−4.3^d^ (−5.1 to −3.5)	2009–2018	−1.4^d^ (−2.3 to −0.5)
≥85	3,694 (8,612.5)	2,820 (5,387.8)	−2.6^d^ (−3.5 to −1.6)	2000–2008	−4.7^d^ (−6.3 to −3.1)	2008–2018	−0.8 (−2.1 to 0.5)
**Race**							
Non-Hispanic Black	3,605 (969.0)	3,169 (617.2)	−2.4^d^ (−2.8 to −2.1)	2000–2012	−3.3^d^ (−3.7 to −3.0)	2012–2018	−0.6 (0.5 to −1.2)
Non-Hispanic White	7,866 (790.0)	6,540 (521.0)	−2.2^d^ (−2.7 to −1.7)	2000–2009	−3.9^d^ (−4.7 to −3.1)	2009–2018	−0.5 (−1.3 to 0.4)
**Race and sex**							
Non-Hispanic Black female	1,975 (846.2)	1,495 (494.5)	−3.0 (−3.4 to −2.1)	2000–2010	−4.1^d^ (−4.6 to −3.5)	2010–2018	−1.6^d^ (−2.4 to −0.7)
Non-Hispanic White female	4,266 (660.9)	3,100 (419.3)	−2.5^d^ (−3.1 to −2.0)	2000–2009	−4.1^d^ (−4.9 to −3.3)	2009–2018	−0.9 (−1.8 to 0)
Non-Hispanic Black male	1,630 (1,137.4)	1,674 (776.2)	−2.1^d^ (−2.7 to −1.5)	2000–2012	−2.9^d^ (−3.5 to −2.3)	2012–2018	−0.5 (−2.2 to 1.2)
Non-Hispanic White male	3,600 (950.5)	3,440 (646.3)	−2.0^d^ (−2.6 to −1.4)	2000–2009	−3.8^d^ (−4.7 to −2.9)	2009–2018	−0.1 (−1.0 to 0.8)

Abbreviation: AAPC, average annual percentage change; APC, annual percentage change.

a Per 100,000 population, adjusted to the 2000 US standard population with age groups 35–54, 55–64, 65–74, 75–84 and ≥85 years.

b The AAPC is a weighted average of the APCs calculated by joinpoint regression.

c The APC is based on age-adjusted rates to the 2000 US standard population.

d AAPC or APC is significantly different (*P* < .05) from 0.

e Per 100,000 population, crude rates.

f Dashes indicate that the best-fit model did not include that trend segment.

### CVD death rates by sex

Among men, the age-adjusted CVD death rate declined from 986.9 deaths per 100,000 population in 2000 to 684.8 deaths per 100,000 in 2018, a relative decline of 30.6% and a significant average annual decline of −2.0% (95% CI, −2.5% to −1.5%). The trends in this group consisted of 2 segments: a significant average annual decline of −3.5%, (95% CI, −4.3% to −2.8%) during the first segment (2000–2009) and a nonsignificant average annual decline in the second segment (2009–2018).

Among women, the age-adjusted CVD death rate declined from 709.6 deaths per 100,000 population in 2000 to 443.4 deaths per 100,000 in 2018, a relative decline of 37.5% and a significant average annual decline of −2.6% (95% CI, −3.1% to −2.2%). In contrast to the findings among men, the trend among women consisted of 2 segments of significant average annual decline in CVD mortality: −4.1% (95% CI, −4.8% to −3.5%) during the first segment (2000–2009) and −1.1% (95% CI, −1.9% to −0.4%) during the second segment (2009–2018) (Table).

### CVD death rates by age

Among adults aged 35 to 54 years, the CVD death rate declined from 132.0 deaths per 100,000 population in 2000 to 125.0 deaths per 100,000 in 2018, a relative decline of −5.3% and a significant average annual decline of −0.5% (95% CI, −0.9% to −0.2%) during only 1 segment (2000–2018). Among adults aged 55 to 64 years, the CVD death rate declined from 513.8 deaths per 100,000 population in 2000 to 396.5 deaths per 100,000 in 2018, a relative decline of 22.8% and a significant average annual decline of −1.1% (95% CI, −1.6% to −0.5%). In this age group, we found 2 segments with contrasting trends: a significant average annual decline of −2.8% (95% CI, −3.4% to −2.2%) in the first segment (2000–2011) and a significant average annual increase of 1.7% (95% CI, 0.6%–2.9%) in the second segment (2011–2018). The CVD death rate among adults aged 65 to 74 years declined from 1,158.3 deaths per 100,000 population in 2000 to 753.1 deaths per 100,000 in 2018, a relative decline of 35.0% and a significant average annual decline of −2.4% (95% CI, −2.9% to −1.9%). The trend in this age group consisted of 2 segments: a significant annual decline of −3.8% (95% CI, −4.3% to −3.3%) in the first segment (2000–2012) and a nonsignificant average annual decline of 0.5% (95% CI, −0.9% to 2.0%) in the second segment (2012–2018). Among those aged 75 to 84 years, the CVD death rate declined from 2,926.3 deaths per 100,000 population in 2000 to 1,701.9 deaths per 100,000 in 2018, a relative decline of 41.8% and a significant average annual decline of −2.9% (95% CI, −3.4% to −2.3%). The trend in this age group consisted of 2 segments: a significant annual decline of −4.3% (95% CI, −5.1% to −3.5%) in the first segment (2000–2009) and a significant annual decline of −1.4% (95% CI, −2.3% to −0.5%) in the second segment (2009–2018). In the oldest age group (≥85 y), the CVD death rate declined from 8,612.5 deaths per 100,000 population in 2000 to 5,387.8 deaths per 100,000 in 2018, a relative decline of 37.4% and a significant average annual decline of −2.6% (95% CI, −3.5% to −1.6%). The trend in this age group consisted of 2 segments: a significant average annual decline of −4.7% (95% CI, −6.3% to −3.1%) in the first segment (2000–2008) and a nonsignificant average annual decline of −0.8% (95% CI, −2.1% to 0.5%) in the second segment (2008–2018).

### CVD death rates by race

Among Black adults in Mississippi, the age-adjusted CVD death rate declined from 969.0 deaths per 100,000 population in 2000 to 617.2 deaths per 100,000 in 2018 (Figure 1 and Table), a relative decline of 36.3% and a significant average annual decline of −2.4% (95% CI, −2.8% to −2.1%). The trend in this group consisted of 2 segments: a significant average annual decline of −3.3% (95% CI, −3.7% to −3.0%) in the first segment (2000–2012) and a nonsignificant average annual decline of −0.6% (95% CI, 0.5% to −1.2%) in the second segment (2012–2018).

Among White adults in Mississippi, the age-adjusted CVD death rate declined from 790.0 deaths per 100,000 population in 2000 to 521.0 deaths per 100,000 in 2018, a relative decline of 34.1% and a significant average annual decline of −2.2% (95% CI, −2.7% to −1.7%). The trend in this group consists of 2 segments: a significant average annual decline of −3.9% (95% CI, −4.7% to −3.1%) in the first segment (2000–2009) and a nonsignificant average annual decline of −0.5% (95% CI, −1.3% to 0.4%) in the second segment (2009–2018).

### CVD death rates by race and sex

**Figure 2:**
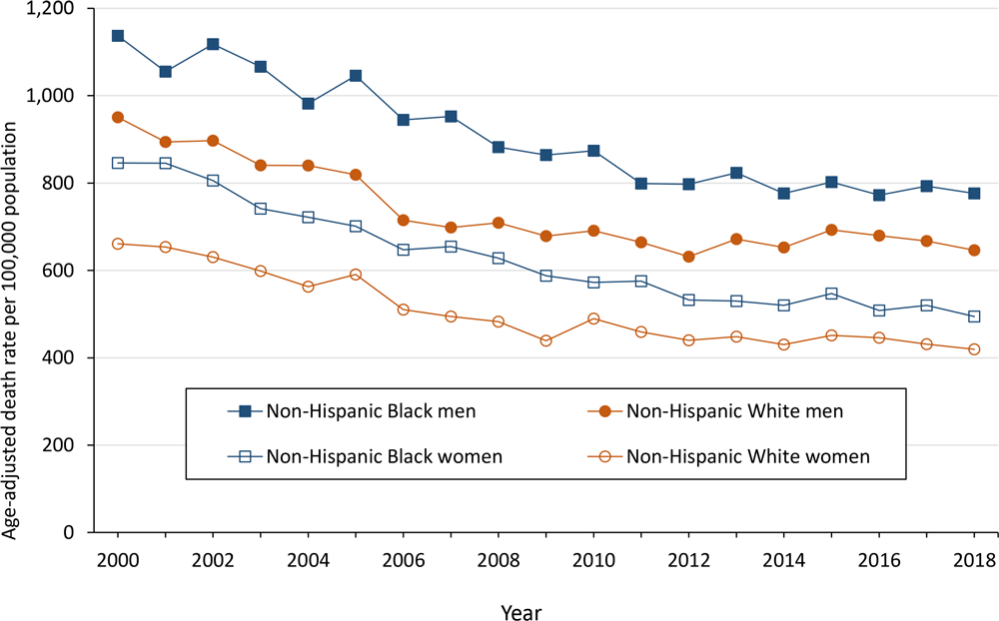
Age-adjusted cardiovascular disease death rates per 100,000 population in Mississippi, by race and sex, 2000–2018.

From 2000 through 2018, the age-adjusted CVD death rate declined among Black women, Black men, White women, and White men in Mississippi (Figure 2). The lowest CVD death rate in 2000 and 2018 was among White women, followed by Black women, White men, and Black men. Among Black women, the age-adjusted CVD death rate declined from 846.2 deaths per 100,000 population in 2000 to 494.5 deaths per 100,000 in 2018, a relative decline of 41.6% and a significant average annual decline of −3.0% (95% CI, −3.4% to −2.1%). The trend in this group consisted of 2 segments: a significant average annual decline of −4.1% (95% CI, −4.6% to −3.5%) in the first segment (2000–2010) and a significant average annual decline of −1.6% (95% CI, −2.4% to −0.7%) in the second segment (2010–2018).

**Table:** 

Year	Death rate per 100,000 population			
	Non-Hispanic Black men	Non-Hispanic White men	Non-Hispanic Black women	Non-Hispanic White women
2000	1,137.4	950.5	846.2	660.9
2001	1,055.4	894.0	845.7	653.7
2002	1,117.8	897.2	806	630.7
2003	1,066.3	840.6	741.5	598.4
2004	982.2	840.4	721.9	562.7
2005	1,046	818.9	701.1	590.9
2006	944.5	715.2	647.4	510.4
2007	952.4	698.5	654.8	494.6
2008	882.5	708.9	628.0	483.0
2009	864.4	678.8	587.8	439.0
2010	874.2	690.9	572.5	489.6
2011	798.9	664.3	575.5	459.1
2012	797.4	631.4	532.4	439.8
2013	823.7	671.6	530.1	448.2
2014	776.1	652.5	520.2	430.2
2015	802.5	693.1	547.3	451.2
2016	772.4	679.7	508.3	445.9
2017	793.1	667.5	520.2	431.2
2018	776.2	646.3	494.5	419.3

Among Black men, the age-adjusted CVD death rate declined from 1,137.4 deaths per 100,000 population in 2000 to 776.2 deaths per 100,000 in 2018, a relative decline of 31.8% and a significant average annual decline of −2.1% (95% CI, −2.7% to −1.5%). The trend in this group consisted of 2 segments: a significant average annual decline of −2.9% (95% CI, −3.5% to −2.3%) in the first segment (2000–2012) and a nonsignificant average annual decline of −0.5% (95% CI, −2.2% to 1.2%) in the second segment (2012–2018).

Among White women, the age-adjusted CVD death rate declined from 660.9 deaths per 100,000 population in 2000 to 419.3 deaths per 100,000 in 2018, a relative decline of 36.5% and a significant average annual decline of −2.5% (95% CI, −3.1% to −2.0%). The trend in this group consisted of 2 segments: a significant average annual decline of −4.1% (95% CI, −4.9% to −3.3%) in the first segment (2000–2009) and a nonsignificant average annual decline of −0.9% (95% CI, −1.8% to 0%) in the second segment (2009–2018).

Among White men, the age-adjusted CVD death rate declined from 950.5 deaths per 100,000 population in 2000 to 646.3 deaths per 100,000 in 2018, a relative decline of 32.0% and a significant average annual decline of −2.0% (95% CI, −2.6% to −1.4%). The trend in this group consisted of 2 segments: a significant average annual decline of −3.8% (95% CI, −4.7% to −2.9%) in the first segment (2000–2009) and a nonsignificant average annual decline of −0.1% (95% CI, −1.0% to 0.8%) in the second segment (2009–2018).

## Discussion

For nearly 2 decades in Mississippi, age-adjusted CVD death rates declined by one-third, with an average annual decline of −2.3%. The analyses demonstrate a relative and annual decline in CVD death rates for all age/race/sex groups in Mississippi. The magnitude of decline, however, varied among subgroups. We observed the lowest rate of decline among adults aged 35 to 64 years and among White women and the highest rate of decline among men, Black adults overall, and Black men.

Our findings contrast with those in a previous study of CVD mortality in Mississippi from 1979 to 1995, which reported an increasing rate of CVD mortality, particularly among Black men (3). Findings from our study are in line with those reported in a nationally representative sample of the US population from 1980 to 2014, which indicated that CVD mortality declined from 507.4 deaths per 100,000 in 1980 to 252.7 deaths per 100,0000, with a relative decline of 50.2% (14). Similarly, Sidney et al reported that all CVD mortality declined in the US between 2011 and 2014 (15). Furthermore, the age-standardized rate of CVD disability-adjusted life-years (DALYs) decreased significantly in Mississippi between 1990 and 2016 (9). Models have shown that the decline in cardiovascular mortality is associated with improvements in both prevention and treatment, including a decrease in cigarette smoking, improved hypertension treatment and control, widespread use of statins to lower cholesterol levels, and the development and timely use of thrombolysis and stents in acute coronary syndrome to limit or prevent myocardial infarction (16,17). For example, in the US between 1980 and 2000, about half the decline in deaths from coronary heart disease were attributable to reductions in major risk factors and the other half to treatment factors (17). In Mississippi, the proportion of current adult smokers declined by 13.9% between 2000 (23.8%) and 2018 (20.5%) (18). In the Mississippi Delta region (a region with a high CVD burden), we found a significant annual decrease in the prevalence of smoking among White adults (APC, −1.99%) (19). Findings from the Jackson Heart Study reported high levels of hypertension treatment (83.2%) and control (66.4%) among Black adults in Mississippi (20).

In addition, we found that age-adjusted CVD death rates did not significantly decrease during the second segment for any group except Black women. Notably, we found a reversal in declining trends among adults aged 55 to 64 years. The increasing prevalence of obesity and diabetes in the US has been posited as contributing factors for these changes (15,18,21). Increases in obesity among this population may be driven by other social determinants of health or unfavorable social conditions, including limited access to nutritious food options and parks for recreation, and household material hardship; these factors are also associated with increases in CVD-related mortality. Prior research suggests that in addition to demographic characteristics, socioeconomic, environmental, and health characteristics account for 60% of the variance in the cardiovascular mortality trajectory (22).

In the US, increases in body mass index (BMI) and the prevalence of diabetes accounted for an 8% and 10% increase, respectively, in coronary heart disease deaths from 1980 to 2000 (17). In Mississippi, dietary risk factors and high systolic blood pressure were the leading risk factors for CVD in both 1990 and 2016, with high BMI becoming a greater contributor and tobacco smoking becoming a lesser contributor to CVD burden during that period (9). In the Jackson Heart Study, obesity defined by waist circumference, waist-to-hip ratio, and BMI, was associated with an increased risk of CVD mortality (23). We previously reported that among adults in Mississippi, the prevalence of obesity (APC, 2.9%) and extreme obesity (APC, 3.6%) increased significantly between 2000 and 2010, with increases occurring across all subgroups (men, women, Black adults, and White adults) (24). Similarly, overweight workers in Mississippi had a 69% higher likelihood of hypertension compared with workers with a normal weight, and the likelihood of hypertension among obese workers was 2.56 times higher (25). In the Jackson Heart Study, diabetes was associated with excess risk for cardiovascular mortality of 2.4% (95% CI, 0.4–4.3) (26). Diabetes prevalence among adults in Mississippi increased by 89.5% between 2000 and 2018, from 7.6% to 14.4% (18).

From 2000 through 2018, the CVD death rate declined for all age groups in Mississippi, and those aged 45 to 64 years had the second lowest magnitude of decline. In an earlier study, we found that among Mississippi workers, the likelihood of having hypertension was significantly higher among those aged 45 to 64 years than among those aged 18 to 29 years (25).

Our finding that the age-adjusted CVD death rate declined for both Black and White adults in Mississippi but was higher among Black than White adults at all time points is similar to previous observations (15,27,28). Trends in both groups consisted of 2 distinct segments: a significant decline in the first segment and nonsignificant decline in the second segment. However, the segments were dissimilar for both groups (9). In Mississippi, the likelihood of hypertension was 19% higher among Black workers than among White workers (25). In the Jackson Heart Study, Black participants who were at high risk of chronic kidney disease incidence or progression who were younger, male, and less educated, and had low levels of trust in health care providers were more likely to report low levels of use of routine medical care (29).

For nearly 2 decades, the age-adjusted CVD death rate declined for both sexes and racial groups in Mississippi. These results align with those reported in other epidemiologic studies (15,27,28). The magnitude of decline was lowest among White and Black men and highest among Black women.

Our study has limitations. First, reliance on death certificates may introduce bias because of the misclassification of the primary cause of death (30). Second, our study used ICD-10 codes, which may be subject to misclassification. A previous study found that the sensitivity and specificity for the underlying cause of death for diseases of the circulatory system was 71.1% and 97.9%, respectively (31). The use of a well-established data set, the analysis of population subgroups using statewide data, and the nearly 2 decades of study are key strengths of the study.

For nearly 2 decades, the overall and age/sex/race-specific CVD death rates significantly declined annually among adults in Mississippi. The magnitude of decline, however, was not uniform across groups or the 2 time segments. The overall rate declined significantly in the first segment (2000–2009), but it did not significantly change annually in the second segment (2009–2018). Although the overall picture of CVD mortality in Mississippi has improved since 2000, our findings identified areas that need attention. Ongoing efforts aimed at monitoring the burden of CVD in Mississippi, such as the Cardiovascular Health Examination Survey in the Mississippi Delta region, are among essential steps to address this need. Moreover, the Mississippi State Department of Health initiatives, such the Mississippi Mayors’ Health Councils program, the Delta Alliance for Congregational Health, and the BROTHERS programs, can play important roles in reducing CVD risk, particularly among adults aged 55-64 years, through tailored approaches.
